# The Mercurial Treatment of Morbus Coxæ

**Published:** 1861-11

**Authors:** W. Godfrey Dyas


ARTICLE IV.

### THE MERCURIAL TREATMENT OF MORBUS COXÆ.

By W. GODFREY DYAS, F.R.C.S.

A case of hip-joint disease has lately presented itself to my notice, which, though not itself remarkable for any special interest, yet recalls to my mind, some points in connection with this form of disease that may be not unprofitably considered. The case is that of a child, five years old, with the right lower extremity, for several months past, deformed, painful and lame. The diseased limb is not merely to appearance, elongated, but it is really so, for on placing the child on his back, and putting the spinous processes of the ilia on the same line, real elongation is manifest. On looking at the patient behind, some curvature of the spine is apparent, but on further examination, it would seem to be caused merely by position. The child has become thin, and his appetite is impaired, but as yet, no serious inroads have been made on his system. When he stands, his right foot is advanced, the leg being slightly flexed on the thigh and the thigh on the pelvis, the toes alone touching the ground. On gently pressing the head of the femur against the acetabulum, or on pressure in the groin over the front of the acetabulum, pain is complained of. Thus far I present the outlines of the case, which may be recognized as one of morbus coxæ. Its features are, as might be supposed, sufficiently marked, to prevent their being confounded with the signs of any other affection; yet, notwithstanding, the case, as I am informed, has been pronounced by some, one of dislocated femur, and this opinion has been accompanied by the recommendation not to interfere with it, owing to its long standing; whilst others, with more correct notions of diagnosis, in recognizing it as morbus coxæ, have been satisfied with restricting their efforts to prescribing the use of Sayre's splints, omitting all other curative measures whatever. It is not my intention to write a formal and methodical treatise on this subject. My object is simply to advocate the claims of a plan of treatment in the cure of this disease,—certainly not to the exclusion of others, but as being that which, in certain stages of this affection, is attended with more beneficial effects than are seen to result from the use of any other internal medicine. This mode of treatment is the mercurial, the existence of which dates from the year 1834. The merit of originality in connection with its introduction, is undoubtedly due to Dr. O'Beirne, one of the Fellows of the College of Surgeons, in Ireland, who, in that year, called the attention of the profession to it as a remedy in white swelling and hip-joint disease. When first published, his views met with the most determined opposition. His opponents saw in the proposal, nothing but the subversion of settled principles, that had already received the sanction of authority. But to this Dr. O'B opposed the result of his experience, and did so with success. The most prejudiced yielded, and mercury became, in the Dublin school, the established practice in the commencement, and likewise in the ulcerative stage of hip-joint disease. It was also adopted in London, and on the continent of Europe, by many eminent surgeons. Amongst others, the celebrated Lisfranc, of Paris, deemed it so important a discovery, that he deputed two distinguished foreigners (Signor Manni and M. Pirondi), to wait on Dr. O'B., to "congratulate him upon having effected such a valuable improvement in the treatment of a disease previously so intractable." In the *Gazette Medicale*, 1845, Lisfranc bears ample testimony to the efficacy of this plan of treatment. "The salivation once set in," he observes, "the pain disappears, as if by magic, and this, perhaps, is one of the therapeutic methods, in which the relation of cause and effect, between the remedy administered and its results is incontestable, on account of the rapidity and constancy with which this result is manifested." But though this mode of treatment has had the sanction of eminent men, it must be conceded that, notwithstanding its striking advantages over other methods, it has not been so generally adopted as it merits, owing, either to surgeons not being aware of its value, or to their previous views being in opposition to it, or perhaps to a feeling of disappointment from having employed it at a period of the disease when it was not only inapplicable, but positively injurious. In employing the mercurial treatment, we must remember there are, at least, two ways of doing anything, and we should, therefore, ascertain accurately beforehand, in what respect the right way differs from the wrong. In view of this, we should commence the treatment at an early period of the disease; and not defer it until the latter shall have advanced to complete disorganization of the joint. This delay would be a misapplication of the proposed means, and would supply an additional instance of what, being a remedy in one stage of a complaint, may be positively injurious in another. The mercurial must be introduced rapidly into the system, not slowly and in alterative doses. The former mode tends to benefit, the latter never fails to impair, the scrofulous constitution. This doctrine may seem strange to some who are wedded to an obsolete dogma of the schools, but it is the doctrine of Graves, one of the most accomplished physicians of modern times. It is admitted by Crampton, by Carmichael, whose anti-mercurial tendencies are well known, and by many others of less note, though men of standing, who possessed in their hospital-practice the fullest opportunities of testing this treatment. Though O'Beirne fully confiding in this remedy when conjoined with rest, sought not the assistance of other means, still the surgeon should not reject any, merely because it is subordinate and secondary. He will, therefore, avail himself of all the aid derivable from local abstractions of blood, by cupping above the trochanter and leeching over the front of the articulation. At a more advanced stage, he will employ blisters and issues. From issues much benefit is often derived. They maintain the ground gained by measures already referred to, and largely contribute to prevent a return of the disease. For many years I have had abundant opportunities of appreciating the importance of this plan of treatment, which I feel confident, is able to cut short the disease in its first stage, to lessen pain and suffering, and to bring about a cure without deformity. It is not to be supposed that success in every case attends its employment. Universal success can scarcely be said to follow any line of treatment in any disease. It is only contended that the mercurial plan of treatment, when conjoined with due attention to rest is more successful than any other in hip-joint disease. But in this, as in other forms of disease, cases may arise that will baffle the best directed efforts of skill, until at length complete disorganization of the structures composing the joint be effected. Even at this stage, when nature fails in her efforts, art still is supposed to have one resource remaining, in the resection or excision of the articulation. This operation, not unknown to the ancients, as may be inferred from some vaguely expressed statement of Hippocrates, has been recommended in more recent times by White of Manchester, and performed by Mr. A. White, of the Westminster Hospital. Mr. Fergusson successfully performed it in 1844, having, as we are told, "meditated its performance for more than ten years, without meeting a single case where it would be justifiable." After its recommendation and performance by British surgeons, the operation was introduced into France by Vermandois and Petit-Radel, who, we are told by Velpeau, "revived this suggestion without modifying it." Velpeau states, that in 1831, he knew of but a single example of exsection of the coxal extremity of the femur practised on living man. But reckoning Mr. A. White's as the first operated on, the next that is seen on record is Mr. Hewson's, of Dublin, operated on in 1823. This case has been published by Leopold (*Uber die Resection*, Wurtzburg, 1834). The result was unsuccessful, the patient having died in three months after the operation. From that date, 1823, there have been twenty-five cases operated on for morbus coxæ, in England; fourteen of which have terminated successfully. It is said, "these operations were all performed in cases where there was no hope of ultimate recovery, and as far as the judgment of the surgeon extended, death would have taken place, had the operation not been performed."—(*Lancet*, 1837.) It has not, however, as yet, generally met the approbation of the profession. The chief objections against it may be summed up under the following heads: It necessitates wounds in diseased parts. It seldom removes the whole disease, but leaves behind degenerated tissues. It is followed by profuse suppuration, and the cure, when effected, is tedious. These objections are over-ruled by the advocates of the measure, who, in deciding on it, are not determined by the pathological condition of the joint, nor by any arbitrary stage of the disease, but propose the operation, if the patient be dying, and there is no hope of saving him by the ordinary means. They contend that the condition of the acetabulum, cannot be ascertained before operation, and that instances have occurred, where it has been successfully executed, although the acetabulum affords evidence of disease at the time of the operation. The particulars of nineteen cases have been published, in every one of which the acetabulum was diseased, in two " there was scarcely a trace of acetabulum. In three the acetabulum was filled with a fibro-gelatinous mass. In six, the gouge was employed for caries. In three it was perforated, and in the others it was more or less affected."—(Hancock *Lancet*, *April*, 1857.) So far from perforation of the acetabulum being a barrier to the operation, Mr. Hancock maintains, that even under these apparently unpromising circumstances, excision may be performed with safety and benefit. The subject under consideration leads me to notice the report of two cases of hip-joint disease, contained in the June number of the MEDICAL EXAMINER. In both, excision of the articulation was performed. The circumstances of each case were unfavorable to a successful operation. In the first, the acetabulum was found to have entirely lost all definite boundaries by absorption of its rim. It was converted into a broad, shallow fossa, and was half filled with granulations. "The head of the femur was carious, denuded of cartilage, and lay with its rough burr-like surface in contact with the flesh on all sides." In concluding the report of the case, it is stated, "that ten days after the operation, it was doing remarkably well." The second case was quite as unpromising as the first. In this, the superior extremity of the femur was excised, and "some suspicious portions of the ilium were removed by the gouge." For eight months after the operation, the patient "was daily passing flatus from the external wound," showing a communication with the rectum, which it was impossible to trace with a probe. Another abscess on the inner side of the thigh having presented itself, and being lanced, gave more ready exit to pus and wind, which were in consequence, transferred to the new opening. In the former case, we are informed of the improvement affected by the operation in ten days after its performance. In the latter, we are merely told, as the result of the operation, "that the opening on the outer side began to heal." It is to be regretted, that these cases have not been more satisfactorily detailed. As they now stand, they are, for all statistical and otherwise practical purposes, positively worthless. To inform us of the condition of the first case in *ten days* after excision, and leave us without further instruction in the matter, cannot enable us to infer anything as to the ultimate result. Ten months would have been more to the point. The probability is, the subject of it, this moment, is beyond the reach of all surgery, conservative or otherwise. The second case constitutes, perhaps, the most formidable form the disease assumes,—that wherein a carious state of the cotyloid cavity exists—-where there is perforation,—communication with the rectum, and pelvic abscess. I am not aware of more than two such cases on record, where recovery resulted. One was under the care of the late Professor Colles. The other was Mr. Hancock's case. In the former, the patient recovered without operation ; in the latter, the femur was excised, and the floor of the acetabulum removed. Now, in the particular case under review, the reader is left to conjecture as he pleases, as to the result. What practical benefit there is in thus publishing cases, it is impossible to see. It only compels the eager inquirer in science, to act the part of Tantalus of old. It does not contribute to the amount of knowledge required to judge of the merits or demerits of a comparatively new operation.

From a comparison of the results, when, on the one hand, the disease is uninterfered with by operative proceedings, and on the other, subjected to resection, I cannot see that much has been gained to science by this operation. If we separate the cases in the last stage of hip joint disease into two classes —those in which the femur is dislocated, lying on the dorsum ilii, and those in which it still continues in the socket—we will find many of the former and a few of the latter recover by the efforts of nature, assisted by such means as directly or indirectly support the system. More than this cannot be said for the operation. A late writer tells us that Moreau, the father, with the hope of removing the carious portions of the cotyloid cavity by means of the chisel and gouge, proposed to two patients, in presence of M. Champion, to perform upon them the operation of the exsection of the femoro-iliac articulation; but they refused and—recovered! This conveys a caution in urging the operation, and will cause the thought to arise, that many, if not all those cases supposed to have been cured by operation, might have done equally well if let alone. I do not altogether adopt this view, but I know it is entertained by some of the most eminent surgeons of the present day.

The question then arises, what cases, if any, are suitable for operation? According to the German Schools, the cases most suitable are the young—those in whom there has been spontaneous luxation, where it is assumed the same amount of mischief has not been effected in the cotyloid cavity as where the head of the femur still remains in its socket in the midst of pus and disorganized tissues. In the former, if the cotyloid cavity be not much affected, its reparative powers, which are considerable, may be called into play, (when not in contact with the diseased femur,) so as to enable it to recover its normal state. On the other hand, in the opinion of Jager, Erichsen and many others, the operation is contra-indicated, where the patient is advanced in years, where the femur remains in the socket, where there are extensive purulent collections in the pelvis or along the course of the femur, and where there is evidence of internal tubercular disease. Unfortunately, the cases in which the femur remains in the socket, are, by a great many, the more numerous, and these are the cases wherein the operation has but little chance of success. But some will not allow any case to be suitable for the operation. Syme condemns it altogether. Erichsen and Jager restrict it to cases which are comparatively few in number, and in which the chance of recovery is, perhaps, as good without, as with the intervention of operative surgery. Brodie speaks as ambiguously as the Delphian Oracle, and says it should be performed "only in those cases where unequivocal advantage may be gained by it." In this unsettled state of professional opinion, perhaps the safest rule of practice that can be deduced from the imperfect data we possess, is not to undertake the operation, unless the constitutional condition of the patient and the incurability of the local affection imperatively require it. Unless, in fact, the patient is dying and there is no other chance. In a word, it should be an operation of *necessity*—not one of expediency.

CHICAGO, October, 1861.

---

REMARKS BY THE EDITOR.—We think the above article of Dr. DYAS scarcely does justice to the subject of the surgical treatment of hip-joint disease. While we fully agree with him, that, in the early stage of the disease, judicious internal, or constitutional treatment is of much importance, we have the most abundant proof, that in all stages, proper surgical appliances and operations can be resorted to with the happiest results. If the writer of the article had made himself as familiar with the medical literature of the last few years, relating to this subject, as he is with the eminent English writers of a little older date, we are quite sure he would not have maintained the doctrine that resection of the head of the femur was to be resorted to *only* as a *forlorn hope.* If he will refer to the published transactions of the *American Medical Association* for 1860, he will find the surgical bearing of the subject very thoroughly discussed; and instead of the twenty-five cases to which he alludes, he will find, carefully tabulated, *one hundred and ten* cases of resection of the head of the femur for caries, the first dating back to 1817.

Of the one hundred and ten cases, thirty-six died, two reported unfavorable, and seventy-two recovered with a more or less useful joint. Neither do we think his strictures on the two cases mentioned in the report of a clinic, by Prof. ANDREWS, published in the June number of the EXAMINER, justly applied. He seems not to have appreciated the fact, that said report was simply the substance of clinical observations, made on cases *then under* treatment, one of which it was expressly stated, had been operated on only "ten days before," and of course, could not yet be reported complete. But we can assure Dr. DYAS, and all others, that the records of these cases are still being kept, and in due time they will be given to the medical public in a form reliable for tabulation. We will simply add, that Case No. 2, to which Dr. DYAS alludes, is still living, looking well and hearty, for we saw her cross the street with only the aid of a cane, almost as quickly as we should, only three days since.

